# The Nation

**Published:** 1867-07

**Authors:** 


THE NATION.—It is with pleasure that we are enabled to call the attention of our readers to such a thoroughly independent and candid journal, expressing the most liberal views, and giving conscientious and just criticisms in politics, literature, social and physical sciences, art and popular education. To the scholar, the student, the teacher, and to all professional men it is equally adapted, leading to sound thinking and right living.

---

Physicians will be pleased to learn that they can obtain the photographs of the Presidents and acting Presidents of the American Medical Association. Through the kindness of Messrs. Jeffers & McDonald we have received a set of photographs, (numbering 21) which are elegantly executed, and it is with pleasure that we aunex their card:

TO THE MEDICAL PROFESSION.

The undersigned, through the liberality of Dr. March of this city, in providing the negatives, are now prepared to furnish card photographs of the Presidents and acting Presidents of the American Medical Association, (21 in number,) at the moderate expense of two dollars.

Gentlemen sending an order enclosing two newspaper stamps and $2.00, will meet with a prompt return.

JEFFERS & McDONALD,
519 Broadway, Albany, N.Y.

Albany, July 15th, 1867.

